# Professor Gross

**Published:** 1841-03

**Authors:** 


### PROFESSOR GROSS.

As our Journal is in the interest of the MEDICAL INSTITUTE OF LOUISVILLE, though not aggressive on other schools, we may be allowed, at the end of the first course of lectures by a new professor, to speak of his labors. It is generally known to our readers, that the chair of SURGERY in the Institute became vacant last summer, and was filled by Dr. Gross, late professor of Pathological Anatomy in the Cincinnati College. Having never taught surgery publicly till he entered on his duties in the Institute, all who feel an interest in its character must desire to know in what manner the new professor has acquitted himself. In stating this, we hope to avoid even the *appearance* of exaggeration, and shall, therefore, limit ourselves to the enunciation, that our pupils, without a single exception known to us, are fully, and indeed, eminently satisfied, both with his lectures and operations. The former have been from beginning to end, extempore; and always delivered with great fluency and precision: the latter have been numerous, diversified and instructive, both on the dead and living subject. Among those on the living, we may mention as the more important, the operation for *Club-foot*, by dividing the tendo achilles; that for *Strabismus*, or squinting, performed eight times within the last month; the operation for *Cataract; Lithotomy*, in which a large calculus was extracted from a boy, nine years old, now restored; tying up the *sub-clavian artery*, for a large and threatening *axillary aneurism*, the patient nearly well; lastly, the extirpation of a tumour, of sixteen years growth, and nearly the size of an orange, seated in the front of the os hyoides, in a young lady, who is recovering. Most of these operations, with many others of a minor character, were performed in presence of the class; either in the amphitheatre of the Institute, or the new clinical and surgical hall of the Louisville Hospital.

We are happy to say, that Professor Gross is now in the act of removing to Louisville, having made his conditional acceptannce permanent; and that he intends henceforth to devote himself to the practice of SURGERY. He will also, hereafter, be a regular contributor to the surgical department of our Journal, which will, indeed, be placed under his special superintendence.

